# PS-29 - VACCINE ELIGIBILITY IN SEVERE COVID-19 AND INFLUENZA: EARLY FINDINGS FROM SCOTLAND

**DOI:** 10.1093/pubmed/fdag046.069

**Published:** 2026-07-09

**Authors:** Mr Matthew Hoyle, Ariadni Kouzeli, Dr Kirstin Leslie, Dr Kirsty Morrison, Dr Josie Evans, Safraj Hameed, Dr Kimberly Marsh

**Affiliations:** Public Health Scotland; Public Health Scotland; Public Health Scotland; Public Health Scotland; Public Health Scotland; Public Health Scotland; Public Health Scotland

## Abstract

**Background:**

Hospital admissions caused by respiratory infections place the Scottish healthcare system under increased pressure during winter months. Vaccination programmes against COVID-19 and seasonal influenza aim to mitigate this impact by offering people vaccines who are at highest risk of severe outcomes, namely those in specific age groups or who have underlying health conditions.

**Objectives:**

To determine the number and proportion of influenza- and COVID-19–related admissions and deaths among vaccine-eligible people, to inform whether eligibility criteria remain appropriate.

**Methods:**

Using anonymised individual–level data via the Scottish Infectious Respiratory Surveillance Platform (SIRSP), we identified and linked hospital admissions with COVID-19 and influenza ICD-10 codes from Scottish Morbidity Records (SMR01) with mortality data from National Records of Scotland (NRS); and vaccine eligibility cohort data for the winter 2024/2025 season (Sept-Mar). Using these routine data, we calculated the proportion of hospital admissions and deaths in people who were vaccine-eligible.

**Results:**

During winter 2024/2025, 2,321 COVID-19 and 7,194 influenza admissions were identified, of which 86% and 82% were eligible for vaccination respectively. Deaths were identified for 178 COVID-19 and 475 influenza admissions. Nearly all (99% for COVID-19 and 97% for influenza) occurred in the eligible population. Of vaccine-ineligible COVID-19 and influenza admissions, age and sex distributions were in line with population averages.

**Conclusions:**

The small proportion of deaths among vaccine-ineligible individuals is reassuring, suggesting current eligibility criteria effectively cover groups at the highest risk of death. Further investigation is required to characterise the demographics and risk profiles of the ineligible admissions to hospital. Linking to comorbidities data collected from NHS clinical patient records, including GP and prescribing data, may help to understand any underlying risk among vaccine-ineligible admissions. Continued monitoring is recommended to understand whether changes to vaccine eligibility criteria remain protective.

